# Utilization of electromyographic biofeedback in speech therapy to attenuate the signs of facial aging: a case report

**DOI:** 10.1590/2317-1782/20232021161en

**Published:** 2023-06-02

**Authors:** Yasmin Salles Frazão, Silvia Bertacci Manzi, Lilian Krakauer, Giédre Berretin-Felix

**Affiliations:** 1 Programa de Pós-graduação em Fonoaudiologia, Faculdade de Odontologia de Bauru, Universidade de São Paulo - USP - Bauru (SP), Brasil.; 2 Conselho Federal de Fonoaudiologia - CFFa - São Paulo (SP), Brasil.; 3 Pontifícia Universidade Católica de São Paulo - PUCSP - São Paulo (SP), Brasil.; 4 Departamento de Fonoaudiologia, Faculdade de Odontologia de Bauru, Universidade de São Paulo - USP - Bauru (SP), Brasil.

**Keywords:** Esthetics, Speech, Language and Hearing Sciences, Myofunctional Therapy, Electromyography, Rejuvenation, Aging

## Abstract

Myotherapy intervention in facial aesthetics aims to attenuate wrinkles and signs of facial aging. The relationship between accentuated muscle contraction during chewing, swallowing and speaking and the appearance of facial wrinkles has been suggested by speech-language pathology literature. This study aimed to report the effect of electromyographic biofeedback associated with training of chewing, swallowing, and smiling patterns, during speech therapy intervention aimed at reducing the signs of facial wrinkles and furrows, in a 55-year-old woman. The therapy also included isotonic and isometric exercises and clinical procedures to decrease the contraction of facial mimicry muscles, which were not associated with training using electromyographic biofeedback. Signal collection and training were performed using the Biotrainer software on the New Miotool Face by Miotec, over nine weekly sessions. Two assessments were performed (before and after the nine sessions), using the MBGR Protocol (assessment of chewing, swallowing, and smiling) and validated scales described in the literature (assessment of signs of facial aging). In the reported case, the usefulness of electromyographic biofeedback was verified for learning trained orofacial myofunctional patterns, as well as to improve chewing and swallowing functions and decrease signs of facial aging. However, further research is needed to demonstrate the positive effects of electromyographic biofeedback associated with myofunctional therapy aimed at attenuating the signs of facial aging.

## INTRODUCTION

The appearance of wrinkles and signs of facial aging can be related to genetic factors and changes that occur in facial structures, such as reabsorption of facial bones, reduction in facial fat and increased contraction of mimicry muscles^(1)^. Additionally, other intrinsic and extrinsic factors contribute to lost skin elasticity and appearance of facial wrinkles and furrows. Intrinsic aging results from physiological and histological changes such as reduced thickness and variation in size and shape of epidermal cells, reduction in fibroblasts, reduction in melanocytes and a reduction in collagen^(1)^. Extrinsic aging is caused by environmental factors such as conditions of hydration, nutrition, smoking, stress, sleep deprivation, and mainly ultraviolet radiation (UV), which leads to photoaging^(1)^.

In addition to extrinsic and intrinsic aging, inadequate facial muscle contractions, performed when chewing, swallowing, and speaking, can produce static and/or dynamic wrinkles, in periorbital, perioral, frontal, and cervical areas, more or less evident depending on the intensity, frequency and duration of the contractions and on individual dentoskeletal characteristics^(2,3,4)^. More studies are necessary however, to demonstrate this correlation frequently described by speech therapists.

The speech therapy proposal to reduce wrinkles and signs of facial aging is a non-invasive therapeutic intervention, with numerous clinical resources being used, including isotonic and isometric exercises, face muscle stretches and orofacial function conditioning^(2,3)^. Surface electromyography (SE) or electromyographic biofeedback remains one clinical resource little investigated by speech therapists in this area.

SE makes functional neuromuscular analysis possible, being considered a valuable tool for both diagnosis and rehabilitation of individuals with orofacial myofunctional alterations^(5)^. Electromyographic biofeedback has been used in association with various speech therapy techniques, to rehabilitate patients with central and peripheral neurological damage, dentoskeletal alterations, and respiratory, voice and functional disorders^(5,6)^. However, publications that report on electromyographic biofeedback used in speech therapy are scarce. Research presenting greater methodological rigor to obtain positive results when using these resources is necessary^(5,6)^.

In the area of orofacial motricity, Rosell-Clari^(7)^ observed that electromyographic biofeedback associated with traditional myofunctional therapy favored control of orofacial muscles while chewing and swallowing, in a 10-year-old child, who presented dentoskeletal and myofunctional alterations. In the only publication regarding the use of electromyographic biofeedback in speech therapy for facial aesthetics, Bernardes^(8)^ described the positive effects of this resource on reducing muscle contraction involved in chewing, swallowing and speech functions, and consequently on reducing facial wrinkles.

As such, the aim of this study was to present the effects of using electromyographic biofeedback associated with functional training of chewing, swallowing, and smiling, during speech therapy interventions seeking to reduce signs of facial aging.

## PRESENTATION OF THE CLINICAL CASE

The present study is a clinical case, associated with the research project approved by the Ethics Committee of the Bauru School of Dentistry - University of São Paulo, reference number 2235918-CAAE: 71680017.0.0000.5417. The female client was duly informed of the aims and procedures to be adopted in the study and signed the Free and Informed Consent.

The client decided to participate in the study due to wanting to undergo a non-invasive treatment to reduce facial wrinkles, having met the following exclusion criteria: realization of invasive facial procedures (plastic surgery, facial filler, botulinum toxin application, laser application) and non-invasive (drainage; massages; medication; new creams, different to those routinely used) in the year prior to the treatments and during participation in the research, a history of skeletal dentofacial deformity, absence of temporomandibular dysfunction, presence of snoring, absence of more than one dental element.

A speech therapy evaluation was performed before and after the therapeutic intervention. The same physical space, equipment, patient position and room lighting were used to collect the images, as recommended by Frazão and Manzi^(9)^. The aesthetic and orofacial myofunctional aspects were analyzed using photographic documentation and in video^(9)^, by two previously trained speech therapists, specialists in orofacial motricity, applying the Orofacial Myofunctional Evaluation Protocol MBGR and the validated scales described in the literature^(10-13)^. Following the initial evaluation, we performed nine sessions undertaking electromyographic biofeedback training associated with chewing, swallowing and mild smiling without labial sealing. During this period, isometric and isotonic exercises were also realized in therapy, to condition the cheek, tongue and suprahyoid muscles (3 series of 20 seconds, using the Facial Plus and Lingual-Pró-Fono^R^ exercisers), the palpebral portion of the orbicularis oculi muscle (3 series of 5 seconds, asking the client to open her eyes wide and hold them open, holding the palpebral portion of the orbicularis oculi muscle firmly) and a speaking exercise with a wine cork between the teeth (opening held at the diameter of the cork - requesting the production of three phrases), as a technique for pronunciation (“speaking with cork”). The client was asked to carry out these exercises daily at home (eye exercises, twice a day, facial and lingual exercisers and “speaking with cork”, once a day), as well as using a Transpore^TM^ inelastic bandage^(3)^ once a day while sleeping at night, over the frontal portion of the right and left occipitofrontal muscle, of the right and left eyebrow corrugator muscle and over the inferior orbital portion of the orbicularis oculi muscle (external corner of the eyes - right and left) and during the day, a small strip of latex (2cm long and 5mm in diameter) in the oral vestibule (intersection between the mentalis muscle and the lower lip) except at meals and while sleeping.

During the electromyographic feedback training we used the Biotrainer software on the New Miotec Miotool Face USB NM600FO device. It has eight channels connected to differential active sensors, with 16bit resolution, a 2Khz sampling frequency, 20Hz low pass filter, 500Hz high pass filter, 60Hz notch, a clamp connection, and a reference electrode (ground). To capture the electric signal, we used the same device with Double Trace LH-ED4020 differential passive dual electrodes, with dimensions 44 mm long, 21 mm wide, and 20 mm from center to center, placed over the muscles engaged when chewing, swallowing, and smiling (right and left masseter muscles; major, minor zygomatic region, risorius muscles; orbicularis oris (upper lip); suprahyoids; inferior orbital portion of the orbicularis oculi muscle). The reference electrode (ground) was positioned over the styloid process of the ulna of the right arm. The facial areas and regions where we positioned the electrodes were sterilized with cotton wool soaked in alcohol 77% GL (70% INPM) prior to placing the electrodes for all sessions when electromyographic biofeedback was employed.

In the initial evaluation, no alterations to chewing function were observed (a score of zero out of 10, for the MGBR Protocol). For swallowing, a marked contraction of the orbicularis oris and mentalis muscles, during routine swallowing of chewed, solid food and guided swallowing of liquid was observed (score 8 out of 28). The analysis of signs of facial aging showed an absence of static perioral wrinkles (score zero), moderate dynamic perioral wrinkles (score two out of four), moderate nasolabial grooves (score two out of four) and mild labiomentonian grooves (score one out of four). The smile evaluation score obtained using video image capture of the dynamic periorbital wrinkles, in the third execution of the “open smile” and of the photographic image of the static periorbital wrinkles in diagonal position (45º), showed accentuated dynamic periorbital wrinkles (score of three out of four) and moderate static periorbital wrinkles (score of two out of four).

**Figure 1:**
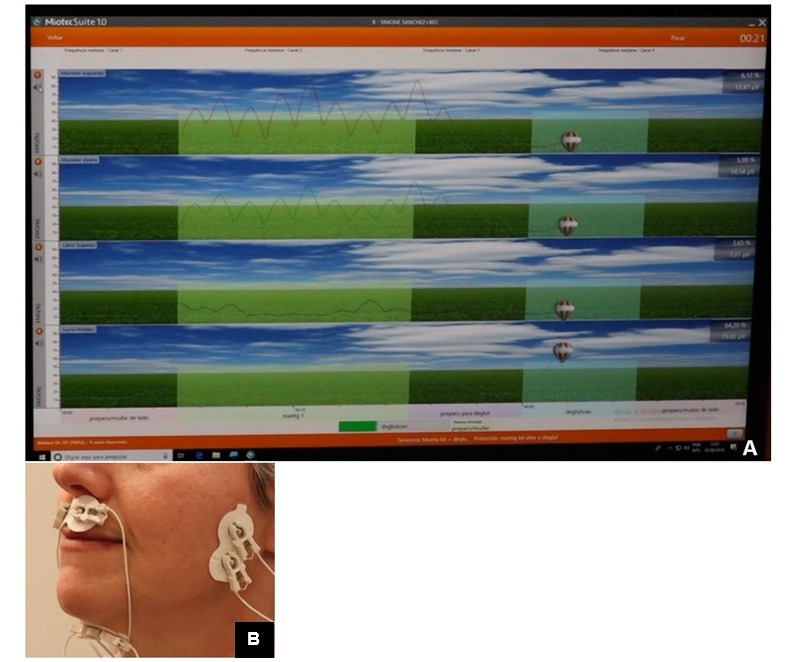
Example of training chewing and swallowing with electromyographic biofeedback and the placement of the electrodes. A - screen with image representative of alternating bilateral chewing and swallowing with muscle tracing masseter, orbicularis oris and suprahyoid muscles; B - electrodes positioned on the masseter, orbicularis oris and suprahyoid muscles

For training with electromyographic biofeedback, the electrodes were placed in a sequence favoring progressive control by the client of the movements of the different muscle groups when executing trained orofacial functions. As such, to train alternating unilateral chewing in the second session, the electrodes were positioned parallel to the direction of the muscle fiber, over the right and left masseter muscles. For the third and fourth sessions, the electrodes were placed over these muscles as well as over the orbicularis oris muscle (upper lip). This position was maintained for the fifth to the ninth sessions, to train alternating bilateral chewing. To train the swallowing function, initiated in the third session with swallowing pasty food (Greek yogurt), the electrodes were initially placed over the suprahyoid muscle region; in the fourth and fifth sessions electrodes were placed over the orbicularis oris muscle (upper lip); these electrodes were maintained to train liquid swallowing in the fifth session, and chewed solid food in the sixth session, when an electrode over the right and left masseter muscles was added and kept until the ninth session. Figure 1B shows the position of the electrodes for training of chewing and swallowing functions. For smile training initiated in the fifth session, an electrode was placed over the region of the risorius muscle and, for the sixth to the ninth sessions, an electrode on the lower orbital portion of the orbicularis oculi muscle was added. Despite placing the electrodes in the risorius region, the possibility of capturing an electric signal from the adjacent muscles cannot be overlooked (cross talk). Even so, during training it was possible to compare the contraction of the risorius region muscles and the inferior orbital portion of the orbicularis oculi muscle. As such, the client managed to reduce the muscle contractions in the eye region while smiling, with consequent reduction of static and dynamic periorbital wrinkles.

Figure 1 shows an example of the use of electromyographic biofeedback and the positioning of electrodes to train chewing and swallowing functions. In the image on the computer screen the client could see the target-line (light blue and green stripes) and the continuous line representing her control over recruitment of the right and left masseter muscles, and orbicularis oris muscles (upper lip) and suprahyoid in chewing (light green stripe) and swallowing (light blue stripe). The continuous line showed that rhythmic activity occurred in the masseter muscles while chewing (two upper stripes on the screen), with little activation of the orbicularis oris muscle (third stripe on the screen - upper lip) and nearly no recruitment of the suprahyoid muscles (third stripe on the screen). For swallowing, only recruitment of the suprahyoid muscles was observed (balloon above the target-line.

The gradual training sequence performed using electromyographic biofeedback in therapy is found in Chart 1.

**Chart 1:** Sequence of functional exercises performed in therapy with electromyographic biofeedback

**AIM SESSIONS**	2^nd^ session	3^rd^ session	4^th^ session	5^th^ session	6^th^ session	7^th^ session	8^th^ session	9^th^ session
alternated unilateral chewing pattern	x	x	x					
bilateral altered chewing pattern				x	x	x	x	x
pasty food swallowing		x	x	x				
liquid swallowing				x	x	x	x	x
solid food swallowing					x	x	x	x
“social smile”				x	x	x	x	x

Maximal voluntary contraction (MVC) of the selected muscles was verified using the Biotrainer software and a 50% MVC value was the parameter established to increase or reduce muscular contraction during training, seeking to attenuate contraction of masseter, orbicularis oris (upper lip), zygomaticus major and minor, and orbicularis oculi muscles, and increase contraction of the levator palpebrae superioris and suprahyoid muscles. The percentage of muscle contraction intensity can be determined using the software, in the Collection Configuration, Protocols tab, Activities tab, New Activity window, intensity (%). The client was instructed to increase muscle contraction to go above the target line or reduce muscle contraction, remaining below the target line.

The client remained seated on her hamstrings, with her feet supported on the ground or on a support stool, maintaining an angle of 90º in the hips, knees, and ankles to perform the electromyographic biofeedback for therapy. The following foods were used to engage swallowing and chewing functions: Greek yogurt, water, and raisins. For the smile, the participant was instructed to smile lightly without lip sealing, while she thought about something happy, keeping her eyes wide open.

Training protocols were created using the Biotrainer software, allowing protocol configuration wherein the activity (chewing, swallowing, smiling, resting) and its duration (in the Collection Configuration window, protocols tab, activities tab) could be indicated. The activities were then inserted into the Protocol Timeline. The training duration for each orofacial function using electromyographic biofeedback was determined by the therapist (author of the present article), producing the following description: alternate unilateral chewing, duration of two minutes and 30 seconds, repeated twice in each session; altered bilateral chewing, duration of one minute and 40 seconds, repeated twice in each session in which this chewing pattern was trained until the conclusion of the program; swallowing of pasty food, duration of one minute and 40 seconds, repeated three times, in the two sessions in which this pattern was trained; swallowing of liquid, duration of one minute and 40 seconds, repeated twice in the first two sessions when this pattern was trained and repeated once a session until the end of the program following this; swallowing of solids (realized at the same time as the training of alternated bilateral chewing), duration of one minute and 40 seconds, repeated twice a session until the end of the program; smile, one minute duration, repeated twice in each session in which this pattern was trained until the end of the program. The client was instructed to control the chewing, swallowing, and smiling patterns at home, in line with the activities performed during therapy.

The benefits of functional training were observed in the scores obtained from MBGR and through the analysis of signs of facial aging after nine sessions performed over nine weeks. The scores obtained in the MBGR were the following: for chewing, the initial score was maintained (score zero out of 10); for swallowing, the score was lower (score two out of 28), with reduced mentalis muscle contraction in guided swallowing of liquid and routine solids swallowing; reduced orbicularis oris muscle contraction for swallowing liquid, without change for swallowing solid food. For signs of facial aging, resulting from excessive contraction of perioral muscles during chewing and swallowing functions, there was an increase in static perioral wrinkles (score one out of four) and reduction of dynamic perioral wrinkles (score of one out of four), of the nasolabial grooves (score of one out of four) and labiomentonian grooves (score of zero out of four). For periorbital wrinkles, which result from exaggerated contraction of the orbicularis oculi muscles while smiling, the score for dynamic periorbital wrinkles remained the same (score of three out of four) and there was a reduction of static periorbital wrinkles (score of one out of four).

**Figure 2:**
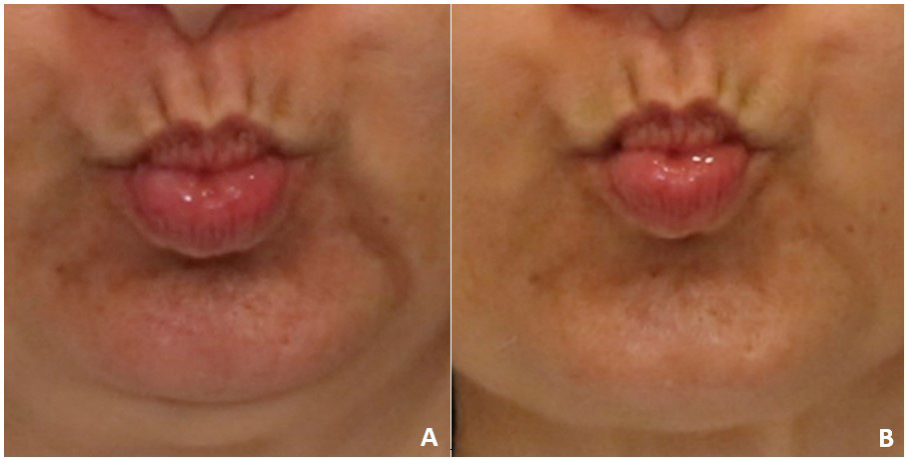
Dynamic perioral wrinkles. A - initial evaluation - moderate wrinkles (score 2 out of 4); B - final evaluation - light wrinkles (score 1 out of 4)

**Figure 3:**
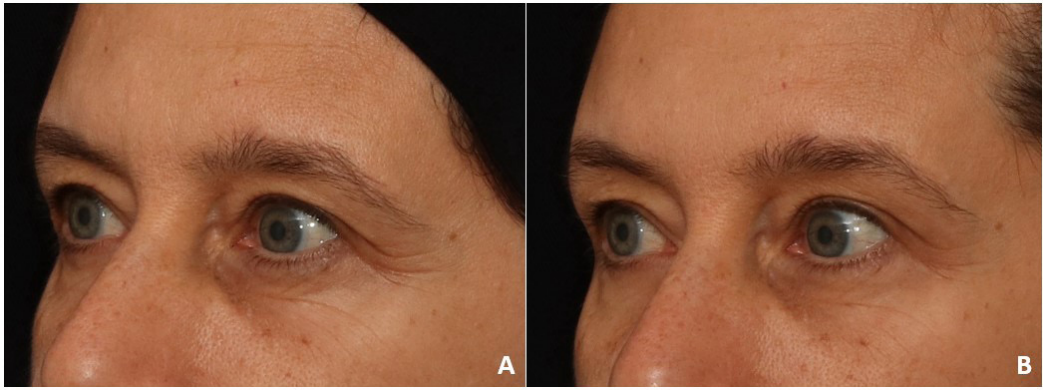
Static periorbital - left diagonal position. A - initial wrinkle evaluation - moderate wrinkles (score 2 out of 4); B - final evaluation - light wrinkles (score 1 out of 4)

**Figure 4:**
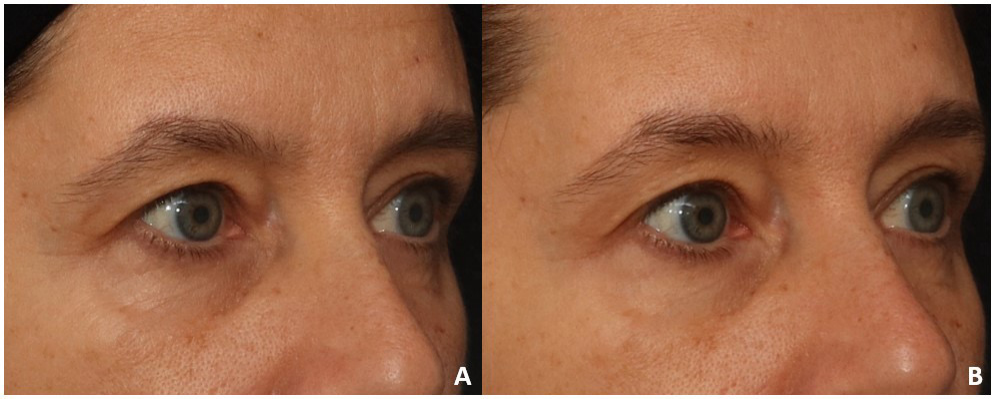
Static periorbital wrinkles - right diagonal position. A - initial wrinkle evaluation - moderate wrinkles (score 2 out of 4); B - final evaluation - light wrinkles (score 1 out of 4)

**Figure 5:**
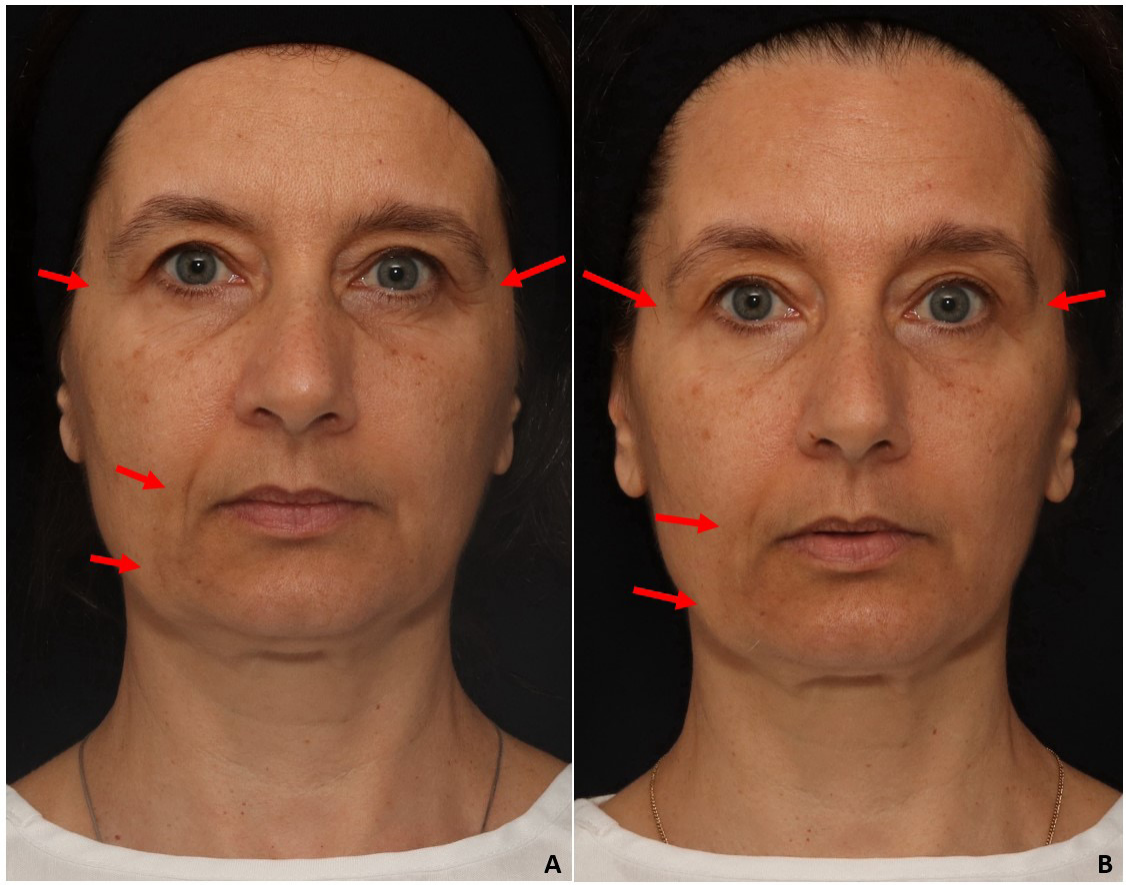
Static periorbital wrinkles - nasolabial groove - labiomentonian groove - frontal position. A - initial evaluation - moderate static periorbital wrinkles (score 2 out of 4) - moderate nasolabial groove (score 2 out of 4) - light labiomentonian groove (score 1 out of 4); B - final evaluation - light static periorbital wrinkles (score 1 out of 4) - light nasolabial groove (score 1 out of 4) - absent labiomentonian groove (score zero out of 4)

Aesthetic changes were observed in the photos and videos (screen capture) performed in the initial and final evaluations, according to the validated scale parameters described in the literature^(10-13)^; dynamic perioral wrinkles in the frontal position (Figure 2), static periorbital wrinkles in the left diagonal position (Figure 3), static periorbital wrinkles in the right diagonal position (Figure 4), static periorbital wrinkles and nasolabial and labiomentonian grooves in the frontal position (Figure 5).

## DISCUSSION

This study aims to present the effects of using electromyographic biofeedback when training chewing, swallowing, and smiling functions, during speech therapy interventions focused on reducing signs of facial aging, in a 55-year-old woman. Positive results from the use of electromyographic biofeedback associated with speech therapy treatments have been described in some studies^(6)^. However, in the field of speech therapy only one publication was observed with a description of three examples in which electromyographic biofeedback was found to be effective to increase awareness and reduce contraction of the muscles involved in chewing, swallowing and speaking. We believe that these satisfactory results need to be consolidated in future studies^(8)^.

The correlation between the presence of wrinkles and signs of facial aging and excessive contraction of mimicry muscles and those used for chewing and swallowing has been described by dermatologists and speech therapists^(1-3)^. In the present study we observed static and dynamic wrinkles on the client’s face, who, while not contracting the perioral musculature excessively while chewing, presented intense contraction of the orbicularis oris and mentalis muscles during swallowing (liquid and solid) and of the orbicularis oculi muscle when smiling and speaking. In the first session, the client was surprised when she looked at her photographs and the video recorded in the initial session. This shows that she was unaware of her oral motor habits, presenting exaggerated orofacial muscle contraction, and was also unaware of the correlation of this with the emergence of facial wrinkles. This understanding was important to increase awareness of altered orofacial movements and for proprioception of adequate orofacial motor patterns to be learnt.

Adequacy of orofacial functions was one of the procedures proposed by speech therapists to attenuate wrinkles and signs of facial aging^(2,3)^. In the second session, training with electromyographic biofeedback to condition chewing, swallowing, and smiling habits was initiated. Using the visualization of muscular activity, with a corresponding image on the computer screen, the client participated more actively and with greater control over her orofacial muscles, being one of the advantages cited by researchers, when using electromyographic biofeedback^(14,15)^.

The placement and number of electrodes used for training can vary depending on therapeutic aims. As such, electrodes can only be positioned on the suprahyoid muscles when training swallowing^(15)^, on the suprahyoid and sternocleidomastoid muscles in alternating voice therapy sessions^(7)^, and on the masseter and orbicularis oris muscles in therapy focusing on facial aesthetics^(8)^. In the present study electrode placement was performed in a sequence favoring the client’s progressive control, giving her awareness and proprioception of the adequate recruitment of the involved muscles to the extent that electrodes were added. Training of alternated unilateral chewing was initiated by the control of masseter movement and subsequently, of the orbicularis oris muscle (upper lip). For swallowing training, in addition to the electrode placement sequence, initially on the suprahyoid muscles and subsequently on the orbicular oris and masseter muscles, there was progression of food consistency. Pasty food (Greek yogurt) was initially offered, followed by liquid (room temperature water) and, finally solid food (raisins). For smile conditioning, the control of the risorius and orbicularis oris muscles associated with happy thinking, contributed to a change in initial habits and a reduction of static periorbital wrinkles. The client was instructed to smile with contraction of risorius muscle towards the ears, keeping the eyes well open, thereby contracting the levator palpebrae superioris muscle.

According to the client, viewing the image corresponding to the muscular contraction on the computer screen favored awareness, control and learning of the movements necessary to perform adequate chewing, swallowing, and facial muscle movement, during the sessions and through fixation of these movements at home. The positive impact provided by visual feedback was also reported in another study involving healthy and dysphagic subjects^(15)^.

After nine sessions performed over nine weeks, it was possible to observe functional and aesthetic changes, leading to more harmonious facial muscle movements. We noted reduced values attributed to swallowing and to signs of facial aging. We also observed a reduction in contraction of the orbicularis oris and mentalis muscles while swallowing and reduced nasolabial and labiomentonian grooves and static periorbital wrinkles. Awareness and proprioception of tongue contraction and position during swallowing and of mimicry muscle contraction during smiling and speech, contributed to reduced perioral muscle contraction and attenuated labiomentonian and nasolabial grooves.

The results presented in this study suggest that electromyographic biofeedback improved uptake of more adequate orofacial habits. However, future studies with a larger number of subjects and control group should be carried out, given that currently no studies were found that demonstrated differences between the outcome of interventions performed with and without the association of this therapeutic resource^(6)^.

## CONCLUSION

In the present study, we described the use of electromyographic biofeedback in a speech therapy intervention seeking to reduce signs of facial aging. We noted the beneficial effect of electromyographic biofeedback in learning the trained orofacial myofunctional patterns, and functional and aesthetic improvements, following the realization of nine speech therapy sessions, with a consensus that this technical resource represents a useful tool for therapeutic contexts.
