# Supplementary material for: Utilization of electromyographic biofeedback in speech therapy to attenuate the signs of facial aging: a case report
Source: Codas. 2023 Jun 2;35(3):e20210161. doi: 10.1590/2317-1782/20232021161en (PMC10266800; doi:10.1590/2317-1782/20232021161en)
Supplement: Translation

# Translation: Utilização do *biofeedback* eletromiográfico na terapia fonoaudiológica para atenuar sinais de envelhecimento facial: relato de caso

**DOI:** 10.1590/2317-1782/20232021161pt

**Authors:** Yasmin Salles Frazão, Silvia Bertacci Manzi, Lilian Krakauer, Giédre Berretin-Felix

**Affiliations:** 1 Programa de Pós-graduação em Fonoaudiologia, Faculdade de Odontologia de Bauru, Universidade de São Paulo - USP - Bauru (SP), Brasil.; 2 Conselho Federal de Fonoaudiologia - CFFa - São Paulo (SP), Brasil.; 3 Pontifícia Universidade Católica de São Paulo - PUCSP - São Paulo (SP), Brasil.; 4 Departamento de Fonoaudiologia, Faculdade de Odontologia de Bauru, Universidade de São Paulo - USP - Bauru (SP), Brasil.

**Keywords:** Estética, Fonoaudiologia, Terapia Miofuncional, Eletromiografia, Rejuvenescimento, Envelhecimento

## Abstract

A intervenção mioterápica em estética facial tem como meta atenuar rugas e sinais de envelhecimento facial. A relação entre contração exagerada dos músculos durante a mastigação, deglutição e comunicação oral e aparecimento de rugas faciais é preconizada na literatura fonoaudiológica neste campo de atuação. Este estudo teve por objetivo relatar o efeito da utilização do *biofeedback* eletromiográfico associado ao treinamento dos padrões de mastigação, deglutição e sorriso, durante intervenção fonoaudiológica direcionada à atenuação de rugas e sulcos faciais, em mulher de 55 anos de idade. O programa terapêutico incluiu exercícios isotônicos, isométricos e procedimentos clínicos para diminuir a contração dos músculos mímicos faciais, que não foram associados ao treinamento com *biofeedback* eletromiográfico. A coleta de sinais e o treinamento com o *software* Biotrainer foram realizados com o aparelho New Miotool Face (Miotec) em nove sessões realizadas semanalmente. Foram realizadas duas avaliações (antes e após as nove sessões), por meio do Protocolo MBGR (avaliação da mastigação, deglutição e sorriso) e de escalas validadas descritas na literatura (avaliação dos sinais de envelhecimento facial). No caso descrito, constatou-se o efeito adjuvante do *biofeedback* eletromiográfico no aprendizado dos padrões miofuncionais orofaciais treinados, como também, a melhoria das funções mastigação e deglutição e a diminuição dos sinais de envelhecimento facial. Porém, são necessárias pesquisas mais robustas que comprovem efeitos positivos do *biofeedback* eletromiográfico associado à terapia miofuncional direcionada a atenuar os sinais de envelhecimento facial.

## INTRODUÇÃO

O aparecimento das rugas e dos sinais de envelhecimento facial pode estar relacionado aos fatores genéticos e às mudanças que ocorrem nas estruturas faciais, como a reabsorção dos ossos faciais, a diminuição da gordura facial e o aumento da contração dos músculos mímicos^(1)^. Além destes, outros fatores, intrínsecos e extrínsecos, concorrem para perda da elasticidade da pele e o aparecimento de rugas e vincos faciais. O envelhecimento intrínseco decorre de mudanças fisiológicas e histológicas tais como, diminuição da espessura, variação no tamanho e na forma das células da epiderme, redução de fibroblastos, diminuição de melanócitos e redução de colágeno^(1)^. O envelhecimento extrínseco é causado por fatores ambientais, tais como condição de hidratação, de nutrição, tabagismo, estresse, privação do sono e, principalmente, a radiação ultravioleta (UV), que resulta no fotoenvelhecimento^(1)^.

Além do envelhecimento intrínseco e extrínseco, as contrações inadequadas dos músculos faciais, realizadas nas funções mastigação, deglutição e fala, poderiam resultar em rugas estáticas e/ou dinâmicas, nas áreas periorbitárias, periorais, frontais e cervicais, mais ou menos evidentes, de acordo com a intensidade, a frequência e a duração destas contrações e, também, com as características dentoesqueléticas dos indivíduos^(2,3,4)^. Porém, mais estudos são necessários para comprovar esta correlação frequentemente descrita por fonoaudiólogos.

A proposta fonoaudiológica para atenuar as rugas e sinais de envelhecimento facial seria a intervenção terapêutica não invasiva, sendo diversos os recursos clínicos utilizados com esta finalidade, tais como, os exercícios isométricos e isotônicos, o alongamento dos músculos faciais e a adequação das funções orofaciais^(2,3)^, porém a eletromiografia de superfície (EMGs) ou o *biofeedback* eletromiográfico são recursos clínicos ainda pouco explorados por fonoaudiólogos neste campo de atuação.

A EMGs possibilita uma análise neuromuscular funcional, sendo considerada instrumento valioso tanto para diagnóstico, como para reabilitação de indivíduos com alterações miofuncionais orofaciais^(5)^. O *biofeedback* eletromiográfico tem sido utilizado, associado a várias técnicas fonoaudiológicas, para reabilitação de pacientes com comprometimento neurológico central, periférico, alterações dentoesqueléticas, distúrbios respiratórios, vocais e funcionais^(5,6)^. Porém, as publicações que descrevem a utilização do *biofeedback* eletromiográfico associado à terapia fonoaudiológica são escassas, sendo necessária uma comprovação dos resultados positivos do uso deste recurso por meio de pesquisas que apresentem maior rigor metodológico^(5,6)^.

Na área da Motricidade Orofacial, Rosell-Clari^(7)^ observou que o *biofeedback* eletromiográfico associado à terapia miofuncional tradicional favoreceu o controle dos músculos orofaciais durante a mastigação e deglutição, em uma criança de dez anos de idade, que apresentou alterações dentoesqueléticas e miofuncionais orofaciais. Em uma única publicação sobre o uso do *biofeedback* eletromiográfico na terapia fonoaudiológica em estética facial, Bernardes^(8)^ descreveu os efeitos positivos do recurso para reduzir a contração dos músculos envolvidos nas funções mastigação, deglutição e fala e, consequentemente, diminuir as rugas faciais.

Sendo assim, o objetivo do presente estudo foi relatar o efeito da utilização do *biofeedback* eletromiográfico associado a um treinamento funcional da mastigação, deglutição e sorriso, durante a intervenção fonoaudiológica direcionada à atenuação dos sinais de envelhecimento facial.

## APRESENTAÇÃO DO CASO CLÍNICO

Trata-se de caso clínico, vinculado ao projeto de pesquisa aprovado pelo Comitê de Ética em Pesquisa, sob número 2235918-CAAE: 71680017.0.0000.5417. A cliente do sexo feminino com 55 anos de idade foi devidamente informada sobre os objetivos e procedimentos realizados no estudo e assinou o Termo de Consentimento Livre e Esclarecido.

A cliente decidiu participar da pesquisa por desejar se submeter a um tratamento não invasivo que atenuasse suas rugas faciais, tendo preenchido os seguintes critérios de exclusão: realização de procedimentos faciais invasivos (cirurgia plástica, preenchimento facial, aplicação de toxina botulínica, aplicação de laser) e não invasivos (drenagem; massagens; medicamentos; novos cremes, distintos dos que habitualmente usava) no ano anterior aos atendimentos e durante a participação na pesquisa, histórico de deformidade dentofacial esquelética, ausência de disfunção temporomandibular, presença de ronco, ausência de mais de um elemento dentário.

Uma avaliação fonoaudiológica foi realizada antes e após intervenção terapêutica. A mesma padronização do espaço físico, equipamentos utilizados, posicionamento do paciente e iluminação da sala foi mantida para a documentação das imagens, conforme preconizado por Frazão e Manzi^(9)^. Os aspectos miofuncionais orofaciais e estéticos foram analisados pela documentação fotográfica e em vídeo^(9)^, por duas fonoaudiólogas previamente calibradas, especialistas em Motricidade Orofacial, por meio do Protocolo de Avaliação Miofuncional Orofacial MBGR e de escalas validadas descritas na literatura^(10-13)^. Após a avaliação inicial, foram realizadas nove sessões quando foi efetuado o treinamento com *biofeedback* eletromiográfico associado à mastigação, deglutição e sorriso suave, sem selamento labial. Neste período foram realizados, também em terapia, exercícios isométricos, exercícios isotônicos para condicionar os músculos da região das bochechas, os supra-hioideos, os linguais (3 series de 20 segundos, utilizando os exercitadores Facial Plus e Lingual - Pró-Fono^R^), a porção palpebral do músculo orbicular do olho (3 series de 5 segundos, solicitando à cliente arregalar olhos e fazer resistência à abertura dos olhos, segurando a porção palpebral do músculo orbicular dos olhos) e exercício de falar com rolha de vinho entre os dentes (abertura mantida pelo diâmetro da rolha - solicitação para produção de três frases), como técnica para articulação (“fala em rolha”). A cliente foi orientada a realizar estes exercícios em seu domicilio diariamente (exercícios de olhos, duas vezes ao dia, exercitadores facial, lingual e “fala em rolha”, uma vez ao dia) e, também diariamente, durante o período de sono noturno, usar bandagem inelástica^(3)^ Transpore^TM^ sobre a porção frontal do músculo occiptofrontal direito e esquerdo, do músculo corrugador do supercílio direito e esquerdo e sobre a porção orbital inferior do músculo orbicular do olho (canto externo dos olhos - direito e esquerdo) e durante o dia, um pequeno pedaço de garrote (2cm de comprimento e 5mm de diâmetro) no vestíbulo oral (intersecção entre mentual e lábio inferior), exceto nas refeições e ao dormir.

No treino com *biofeedback* eletromiográfico foi utilizado o software Biotrainer no aparelho *New Miotool Face USB* NM600FO, da Miotec, que possui oito canais conectados a sensores ativos diferenciais, com resolução de 16bits, frequência de amostragem 2Khz, filtro passa-baixa 20Hz, filtro passa-alta 500Hz, notch 60Hz, com conexão de garras e um de referência (terra). Para a captação do sinal elétrico, realizado com o mesmo aparelho, foram utilizados eletrodos duplos passivos diferenciais, Double Trace LH-ED4020; dimensões: 44 mm de comprimento, 21 mm de largura, 20 mm de centro a centro, colocados sobre músculos envolvidos na mastigação, deglutição e sorriso (masseter direito e esquerdo; região de zigomático maior, menor, risório; orbicular da boca - lábio superior; supra-hioideos; porção orbital inferior do músculo orbicular do olho). O eletrodo de referência (terra) foi posicionado sobre o processo estiloide da ulna do braço direito. As áreas da face e a região em que o eletrodo de referência foi colocado foram higienizadas com gaze embebida em álcool 77º GL (70º INPM) em todas as sessões em que o treino com *biofeedback* eletromiográfico foi realizado, antes da colocação dos eletrodos.

Na avaliação inicial, não foram observadas alterações na função mastigação (pontuação zero em 10, no Protocolo MBGR). Em relação à deglutição, constatou-se contração acentuada dos músculos orbicular da boca e mentual, durante deglutição habitual de alimento sólido triturado e deglutição dirigida de líquido (pontuação oito em 28). A análise dos sinais de envelhecimento facial revelou ausência de rugas periorais estáticas (pontuação zero), rugas periorais dinâmicas moderadas (pontuação dois em quatro), sulco nasolabial moderado (pontuação dois em quatro) e labiomentoniano suave (pontuação um em quatro). A pontuação na avaliação do sorriso, obtida por meio da captura da imagem de vídeo das rugas periorbitárias dinâmicas, na terceira execução do “sorriso aberto” e da imagem fotográfica das rugas periorbitárias estáticas na posição diagonal (45º), revelou a presença de rugas periorbitárias dinâmicas acentuadas (pontuação três em quatro) e rugas periorbitárias estáticas moderadas (pontuação dois em quatro).

Para o treinamento com *biofeedback* eletromiográfico, os eletrodos foram colocados em uma sequência para favorecer o controle progressivo da cliente sobre os movimentos dos diferentes grupos musculares na execução da funções orofaciais treinadas. Assim, para o treino da mastigação unilateral alternada, na segunda sessão, os eletrodos foram posicionados, paralelamente ao sentido da fibra muscular, sobre os músculos masseteres direito e esquerdo; na terceira e quarta sessões, os eletrodos foram posicionados sobre estes músculos e, também, sobre o músculo orbicular da boca (lábio superior); este posicionamento foi mantido da quinta à nona sessão, para o treino da mastigação bilateral alternada. Para o treino da função deglutição, iniciado na terceira sessão com a deglutição de alimento pastoso (iogurte grego), os eletrodos foram posicionados, inicialmente, na região dos músculos supra-hioideos; na quarta e quinta sessões foram acrescentados eletrodos sobre o músculo orbicular da boca (lábio superior); estes eletrodos foram mantidos no treinamento da deglutição de líquido, na quinta sessão, do alimento sólido triturado na sexta sessão, quando foi acrescentado o eletrodo sobre músculos masseteres direito e esquerdo e mantidos até a nona sessão. A Figura 1B ilustra o posicionamento dos eletrodos no treinamento das funções mastigação e deglutição. Para o treino do sorriso iniciado na quinta sessão, um eletrodo foi posicionado na região do músculo risório e, da sexta à nona sessão, foi acrescentado um eletrodo na porção orbital inferior do músculo orbicular do olho. Apesar do posicionamento de eletrodos na região do risório, não pode ser descartado a captação do sinal elétrico dos músculos adjacentes (*cross talk*). Ainda assim, durante o treinamento foi possível comparar a contração dos músculos da região do risório e porção orbital inferior do músculo orbicular do olho, isto é, a cliente conseguia reduzir a contração muscular na região dos olhos ao sorrir, com consequente atenuação de rugas periorbitárias estáticas e dinâmicas.

**Figura 1:** Exemplo do treinamento de *biofeedback* eletromiográfico da mastigação e deglutição e o posicionamento dos eletrodos. A - tela com imagem representativa da mastigação bilateral alternada e deglutição com traçado dos músculos masseteres, orbicular da boca e supra-hioideos; B - eletrodos posicionados sobre os músculos masseteres, orbicular da boca e supra-hioideos

A Figura 1 ilustra um exemplo do uso do *biofeedback* eletromiográfico e o posicionamento dos eletrodos no treino das funções de mastigação e deglutição. Na imagem da tela do computador a cliente pode visualizar o traçado-alvo (tarjas verde clara e azul clara) e os traços (linha contínua), representando seu controle sobre o recrutamento dos músculos masseter direito e esquerdo, orbicular da boca (lábio superior) e supra-hioideos na mastigação (tarja verde clara) e deglutição (tarja azul clara). O traço contínuo revelou que durante a mastigação ocorreu atividade ritmada dos músculos masseteres (duas faixas superiores na tela), pouca ativação do músculo orbicular da boca (terceira faixa na tela - lábio superior) e praticamente nenhum recrutamento dos músculos supra-hioideos (quarta faixa na tela). Na deglutição observou-se apenas o recrutamento dos músculos supra-hioideos (balão acima do traçado-alvo).

A sequência do treinamento gradual realizado por meio do biofeedback eletromiográfico em terapia se encontra ilustrada no Quadro 1.

**Quadro 1:** Sequência dos treinos funcionais realizados em terapia com o uso do biofeedback eletromiográfico

**OBJETIVO SESSÕES**	2ª sessão	3ª sessão	4ª sessão	5ª sessão	6ª sessão	7ª sessão	8ª sessão	9ª sessão
padrão mastigatório unilateral alternado	x	x	x					
padrão mastigatório bilateral alternado				x	x	x	x	x
deglutição de alimento pastoso		x	x	x				
deglutição de líquido				x	x	x	x	x
deglutição de alimento sólido					x	x	x	x
“sorriso social”				x	x	x	x	x

A contração voluntária máxima (CVM) dos músculos selecionados foi aferida por meio do *software Biotrainer* e o valor de 50% da CVM foi o parâmetro estabelecido para o aumento ou diminuição da contração muscular durante os treinos, cujo objetivo foi atenuar a contração dos músculos masseteres, orbicular da boca (lábio superior), zigomáticos maior e menor, orbicular do olho e aumentar a contração do músculo levantador da pálpebra superior e supra-hioideos. O percentual da intensidade da contração muscular pode ser estabelecido no *software*, na janela Configuração de Coleta, aba Protocolos, aba Atividades, janela Nova Atividade, Intensidade(%). A cliente foi orientada a aumentar a contração muscular, ultrapassando o traçado-alvo ou reduzir a contração muscular mantendo-se abaixo deste traçado.

A cliente permaneceu sentada sobre os ísquios, com pés apoiados no chão ou banco de apoio, mantendo angulo de 90º em quadris, joelhos e tornozelos para a realização do *biofeedback* eletromiográfico em terapia. Os seguintes alimentos foram utilizados para trabalhar as funções de mastigação e deglutição: iogurte grego, água e uva passa. Para o sorriso, a participante foi orientada a fazer um sorriso suave sem selamento labial, enquanto pensava em algo alegre, mantendo os olhos bem abertos.

Os protocolos de treino foram criados no *software* Biotrainer, que permite a configuração de protocolos nos quais é possível nomear a atividade (mastigação, deglutição, sorriso, repouso) e sua duração (na janela Configuração de Coleta, aba Protocolos, aba Atividades). As atividades são, então, inseridas na Linha do Tempo do Protocolo. A duração do treinamento de cada uma das funções orofaciais utilizando o *biofeedback* eletromiográfico foi determinada pela terapeuta (autora do presente artigo), resultando na seguinte descrição: mastigação unilateral alternada, duração de dois minutos e 30 segundos, repetido duas vezes na sessão; mastigação bilateral alternada, duração de um minuto e 40 segundos, repetido duas vezes em cada sessão em que esse padrão mastigatório foi treinado até a conclusão do programa; deglutição de pastoso, duração de um minuto e 40 segundos, repetido três vezes, nas duas sessões em que este padrão foi treinado; deglutição de líquido, duração de um minuto e 40 segundos, repetido duas vezes nas duas primeiras sessões em que esse padrão foi treinado e repetido uma vez na sessão até o final do programa; deglutição de sólido (realizado no mesmo momento que o treino de mastigação bilateral alternada), duração de um minuto e 40 segundos, repetido duas vezes nas sessões até o final do programa; sorriso, duração de um minuto, repetido duas vezes nas sessões em que esse padrão foi treinado até o final do programa. A cliente recebeu orientações para controlar os padrões de mastigação, deglutição e sorriso, de acordo com o treino realizado em terapia, em sua residência.

Após nove sessões realizadas semanalmente, os escores aferidos no MBGR e na análise dos sinais de envelhecimento facial revelaram os benefícios do treinamento funcional realizado. As pontuações obtidas no MBGR foram as seguintes: na mastigação, o escore inicial foi mantido (pontuação zero em 10); na deglutição, houve redução do escore (pontuação dois em 28), com diminuição da contração do músculo mentual na deglutição dirigida de líquido e deglutição habitual de sólido; diminuição da contração do músculo orbicular da boca na deglutição de líquido, sem mudança para a deglutição de alimento sólido. Em relação aos sinais de envelhecimento facial, que seriam resultantes da contração excessiva dos músculos periorais durante as funções mastigação e deglutição, houve aumento das rugas periorais estáticas (pontuação um em quatro) e diminuição das rugas periorais dinâmicas (pontuação um em quatro), do sulco nasolabial (pontuação um em quatro) e sulco labiomentoniano (pontuação zero em quatro). Em relação às rugas periorbitárias, que seriam resultantes da contração exagerada do orbicular do olho durante sorriso, o escore das rugas periorbitárias dinâmicas manteve-se inalterado (pontuação três em quatro) e houve diminuição das rugas periorbitárias estáticas (pontuação um em quatro).

As mudanças estéticas foram visualizadas nas imagens fotográficas e em vídeo (captura de tela) realizadas nas avaliações inicial e final, de acordo com os parâmetros das escalas validadas descritas na literatura^(10-13)^: rugas periorais dinâmicas na posição frontal (Figura 2), rugas periorbitárias estáticas na posição diagonal esquerda (Figura 3), rugas periorbitárias estáticas na posição diagonal direita (Figura 4), rugas periorbitárias estáticas, sulcos nasolabial e labiomentoniano na posição frontal (Figura 5).

**Figura 2:** Rugas periorais dinâmicas. A - avaliação inicial - rugas moderadas (pontuação 2 em 4); B - avaliação final - rugas suaves (pontuação 1 em 4)

**Figura 3:** Periorbitárias estáticas - posição diagonal esquerda. A - avaliação inicial rugas - rugas moderadas (pontuação 2 em 4); B - avaliação final - rugas suaves (pontuação 1 em 4)

**Figura 4:** Rugas periorbitárias estáticas - posição diagonal direita. A - avaliação inicial - rugas moderadas (pontuação 2 em 4); B - avaliação final - rugas suaves (pontuação 1 em 4)

**Figura 5:** Rugas periorbitárias estáticas - sulco nasolabial - sulco labiomentoniano - posição frontal. A - avaliação inicial - rugas periorbitárias estáticas moderadas (pontuação 2 em 4) - sulco nasolabial moderado (pontuação 2 em 4) - sulco labiomentoniano suave (pontuação 1 em 4); B - avaliação final - rugas periorbitárias estáticas suaves (pontuação 1 em 4) - sulco nasolabial suave (pontuação 1 em 4) - sulco labiomentoniano ausente (pontuação zero em 4)

## DISCUSSÃO

O objetivo do presente estudo foi relatar o efeito da utilização do *biofeedback* eletromiográfico associado ao treinamento dos padrões de mastigação, deglutição e sorriso, durante intervenção fonoaudiológica direcionada à atenuação dos sinais de envelhecimento facial, em mulher de 55 anos de idade. Resultados positivos sobre o uso do *biofeedback* eletromiográfico associado às terapias fonoaudiológicas foram descritos em alguns estudos^(6)^. Porém, no campo da fonoaudiologia em estética facial foi encontrada apenas uma publicação com a descrição de três exemplos em que o *biofeedback* eletromiográfico mostrou-se efetivo para conscientizar e diminuir a contração dos músculos envolvidos na mastigação, na deglutição e articulação da fala. Considerou-se que estes resultados satisfatórios precisam ser consolidados em futuras pesquisas^(8)^.

A correlação entre a presença de rugas e sinais de envelhecimento na face e a contração excessiva dos músculos mímicos e daqueles utilizados na mastigação e deglutição foi descrita por alguns dermatologistas e fonoaudiólogos^(1-3)^. Observou-se, no presente estudo, a presença de rugas estáticas e dinâmicas na face da cliente, que, embora não tenha contraído a musculatura perioral exageradamente durante a mastigação, apresentou contração intensa dos músculos orbicular da boca e mentual na deglutição (líquido e sólido) e do músculo orbicular do olho, no sorriso e na fala. Na primeira sessão, a cliente demonstrou surpresa ao olhar suas fotografias e vídeo registrados na avaliação inicial; revelou que nunca havia percebido seu padrão motor oral, com presença de contração exagerada da musculatura orofacial e desconhecia a correlação entre esta contração e o aparecimento de rugas faciais. Esta percepção foi importante para o trabalho de conscientização dos movimentos orofaciais alterados e de propriocepção do padrão motor orofacial adequado a ser aprendido.

A adequação das funções orofaciais foi um dos procedimentos propostos por fonoaudiólogos para atenuar rugas e sinais de envelhecimento facial^(2,3)^. Na segunda sessão o treinamento com *biofeedback* eletromiográfico, para adequar o padrão de mastigação, deglutição e sorriso, foi iniciado. Por meio da visualização da atividade muscular, representada por imagem correspondente na tela do computador, a cliente pode manter uma participação mais ativa e maior controle sobre sua musculatura orofacial, sendo esta uma das vantagens citada por pesquisadores, para a utilização do *biofeedback* eletromiográfico^(14,15)^.

O posicionamento e número de eletrodos utilizados no treinamento pode variar de acordo com os objetivos terapêuticos. Assim, eletrodos podem ser posicionados apenas sobre os músculos supra-hioideos, durante o treinamento deglutição^(15)^, sobre os músculos supra-hioideos e esternocleidomastóideo, em sessões alternadas de terapia vocal^(7)^, sobre os músculos masseteres e orbicular da boca, na terapia direcionada para a estética facial^(8)^. No presente estudo a colocação dos eletrodos foi realizada em uma sequência para favorecer o controle progressivo da cliente, que adquiriu conscientização e propriocepção sobre o recrutamento adequado dos músculos abordados, à medida que eletrodos eram acrescentados. O treino da mastigação unilateral alternada foi iniciado pelo controle da movimentação dos masseteres e, posteriormente, do músculo orbicular da boca (lábio superior). No treino da deglutição, além da sequência para a colocação dos eletrodos, inicialmente, posicionados sobre os músculos supra-hioideos, em seguida sobre os músculos orbicular da boca e masseteres, houve progressão da consistência do alimento; foi oferecido, inicialmente, alimento pastoso (iogurte grego), em seguida líquido (água em temperatura ambiente) e, finalmente, sólido (uva passa). Na adequação do sorriso, o controle sobre os músculos risório e orbicular do olho associado a um pensamento alegre, contribuiu para mudança do padrão inicial e diminuição das rugas periorbitárias estáticas. A cliente foi orientada a sorrir com contração do músculo risório em direção às orelhas, mantendo os olhos bem abertos, com contração do músculo levantador da pálpebra superior.

Segundo a cliente, a visualização na tela do computador, de uma imagem correspondente a uma contração muscular, favoreceu a conscientização, o controle e o aprendizado de movimentos necessários para a realização dos padrões adequados de mastigação, deglutição e movimentação dos músculos faciais, durante as sessões e a fixação destes padrões em sua residência. O impacto positivo proporcionado pelo *feedback* visual foi também relatado em outro estudo, em que participaram sujeitos saudáveis e disfágicos^(15)^.

Após nove sessões realizadas semanalmente, foi possível constatar mudanças estéticas e funcionais, resultando em movimentação mais harmônica dos músculos faciais. Constatou-se diminuição dos valores atribuídos à deglutição e aos sinais de envelhecimento facial. Observou-se redução na contração dos músculos orbicular da boca e mentual, na deglutição, atenuação dos sulcos nasolabial e labiomentoniano e das rugas periorbitárias estáticas. A conscientização e propriocepção da posição e contração da língua na deglutição e da contração dos músculos mímicos no sorriso e na fala, contribuíram para diminuir a contração dos músculos periorais e atenuar os sulcos nasolabial e labiomentoniano.

Os resultados apresentados no presente estudo sugerem que o *biofeedback* eletromiográfico potencializou o aprendizado de padrões orofaciais mais adequados. Porém, estudos futuros com maior número de sujeitos e grupo controle deverão ser realizados, uma vez que até o presente momento não foram encontrados estudos que comprovem diferenças entre os desfechos finais de intervenções realizadas com e sem a associação deste recurso terapêutico^(6)^.

## CONCLUSÃO

No presente estudo foi descrita a utilização do *biofeedback* eletromiográfico na intervenção fonoaudiológica direcionada à atenuação dos sinais de envelhecimento facial. Constatou-se efeito adjuvante do *biofeedback* eletromiográfico no aprendizado dos padrões miofuncionais orofaciais treinados, melhorias estéticas e funcionais, após a realização de nove sessões de terapia fonoaudiológica, havendo consenso que este recurso técnico representa uma modalidade coadjuvante promissora no processo terapêutico.
